# Cholera

**Published:** 1885-09

**Authors:** 


CHOLERA.

The ravages of cholera in Spain continue, and excite the sympathy of the world.

The experience of last year seems to have been lost in Marseilles, and that city is again paying the penalty that filth and neglect of sanitary measures impose.

It was but natural to suppose that after such afflictions as

Toulon and Marseilles experienced, a year ago, there would have been no neglect of any sanitary measures calculated to prevent a recurrence of that dreadful disease this year, but, if the reports that reach us be true, but little has been done by the authorities of either Toulon or Marseilles to improve the sanitary condition of those cities in the interval, although the fact has been known to them, and to the world, that their condition was of the most favorable character for the spread of the disease. The authorities were indifferent in the interval: now all classes of society seem to be suffering, and reports say the populace are terror-stricken.

It is fortunate that the disease has been confined to so restricted a territory until the season is thus far advanced. This gives timely warning to other countries, having constant intercourse with those thus afflicted, that no precautions should be omitted to prevent introduction of the disease from those countries.

Distance should not lull the United States into fancied security, nor the lateness of the season lead to the relaxing of quarantine regulations nor sanitary measures, especially along the lines of travel and traffic.

It would scarcely seem necessary to remind the authorities and the public that regrets are useless and unavailing when opportunities for securing safety have been thrown away and the plague is in our midst, were it not that Toulon and Marseilles are now offering evidence of the negligence that seems to characterize people, even in the face of threatening danger. With those examples, and the present experience in Spain, it would be culpable if the authorities of the general government of the United States, and our state and local health-officials should omit any part of their important duties in an effort to prevent the introduction and spread of cholera in America.

